# Supplementary material for: Subject-specific whole-brain parcellations of nodes and boundaries are modulated differently under 10 Hz rTMS
Source: Sci Rep. 2023 Aug 3;13:12615. doi: 10.1038/s41598-023-38946-5 (PMC10400653; doi:10.1038/s41598-023-38946-5)
Supplement: Supplementary file 1 — Supplementary Information. [file 41598_2023_38946_MOESM1_ESM.docx]

***Supplementary Material***

**Subject-specific whole-brain parcellations of nodes and boundaries are modulated differently under 10Hz rTMS**

Vladimir Belov^1,#^, Vladislav Kozyrev^1,2,3#^, Aditya Singh^1^, Matthew D. Sacchet^4^, Roberto Goya-Maldonado^1,^*

Affiliations:

^1^ Laboratory of Systems Neuroscience and Imaging in Psychiatry (SNIP-Lab), Department of Psychiatry and Psychotherapy, University Medical Center Göttingen (UMG), Göttingen, Germany

^2^ Functional Imaging Laboratory, German Primate Center – Leibniz Institute for Primate Research, Göttingen, Germany

^3^ Institute of Molecular and Clinical Ophthalmology Basel, Basel, Switzerland

^4^ Meditation Research Program, Department of Psychiatry, Massachusetts General Hospital, Harvard Medical School, Boston, MA, USA

Running title: rTMS modulates nodes and boundaries

**# equal contribution**

***Corresponding author:**

PD Dr. Roberto Goya-Maldonado

Laboratory of Systems Neuroscience and Imaging in Psychiatry (SNIP-Lab)

Department of Psychiatry and Psychotherapy

University Medical Center Göttingen (UMG)

Von-Siebold Str. 5, 37075 Göttingen

e-mail: [roberto.goya@med.uni-goettingen.de](mailto:roberto.goya@med.uni-goettingen.de)


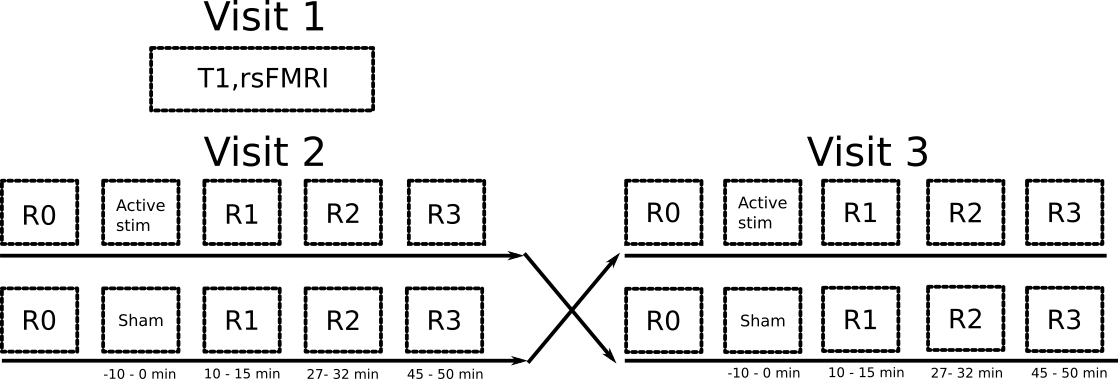


**Supplementary Figure 1**: Study design – We acquired T1 and rsfMRI images at visit 1 that were used for personalized target selection. The found target was then located in T1 image for stimulation one week after (Visit 2) and two weeks after (Visit 3) via online neuronavigation. Each subject was assigned to an arm of the crossover design, receiving both real and sham stimulation in a counterbalanced manner. At the beginning of the sessions on Visit 2 and Visit 3, we obtained a baseline rsfMRI scan (R0). After the 10 Hz rTMS was delivered, three rsfMRI scans (R1, R2 R3) were obtained.


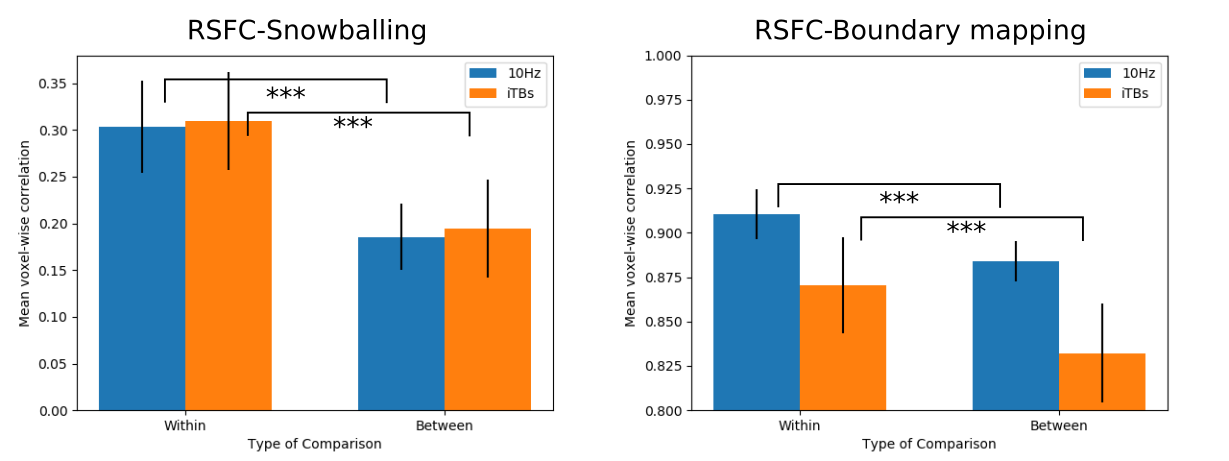
**Supplementary Figure 2**: Validation of 3D maps – Spatial correlation within and between node density maps (left) and boundaries (right) in healthy control subjects. Two datasets of baseline rsfMRI separated by about 1 week from independent cohorts of healthy controls (“10Hz” with 23 subjects and “iTBS” with 26 subjects in blue and orange, respectively). For both cohorts, within subject correlation was significantly higher (***p<0.001) than between subjects correlation in both nodes and boundaries. Bars represent SD.

***
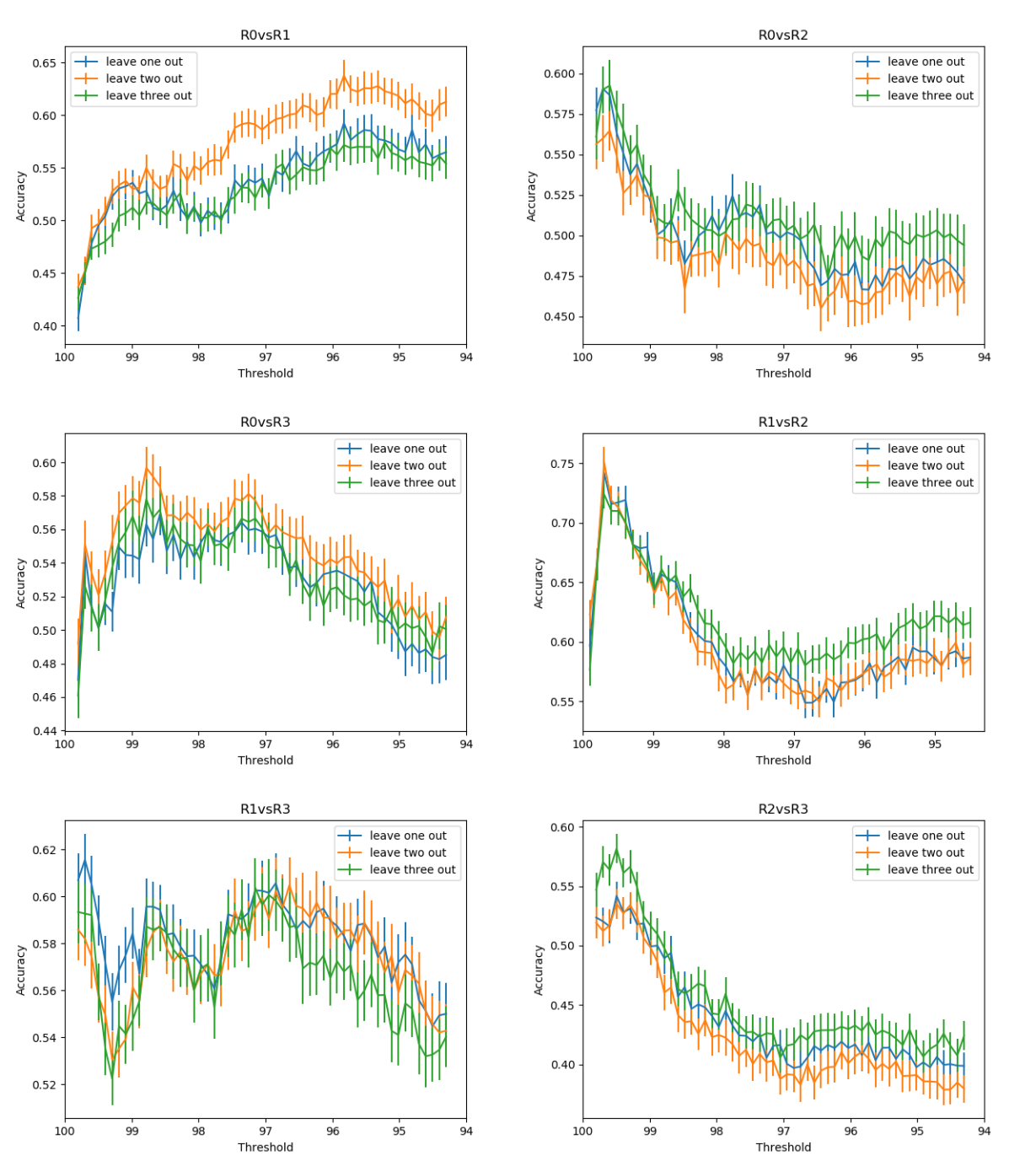
***

**Supplementary Figure 3:** Pairwise classification accuracies of nodes’ density maps using three different cross-validation (CV) strategies

***
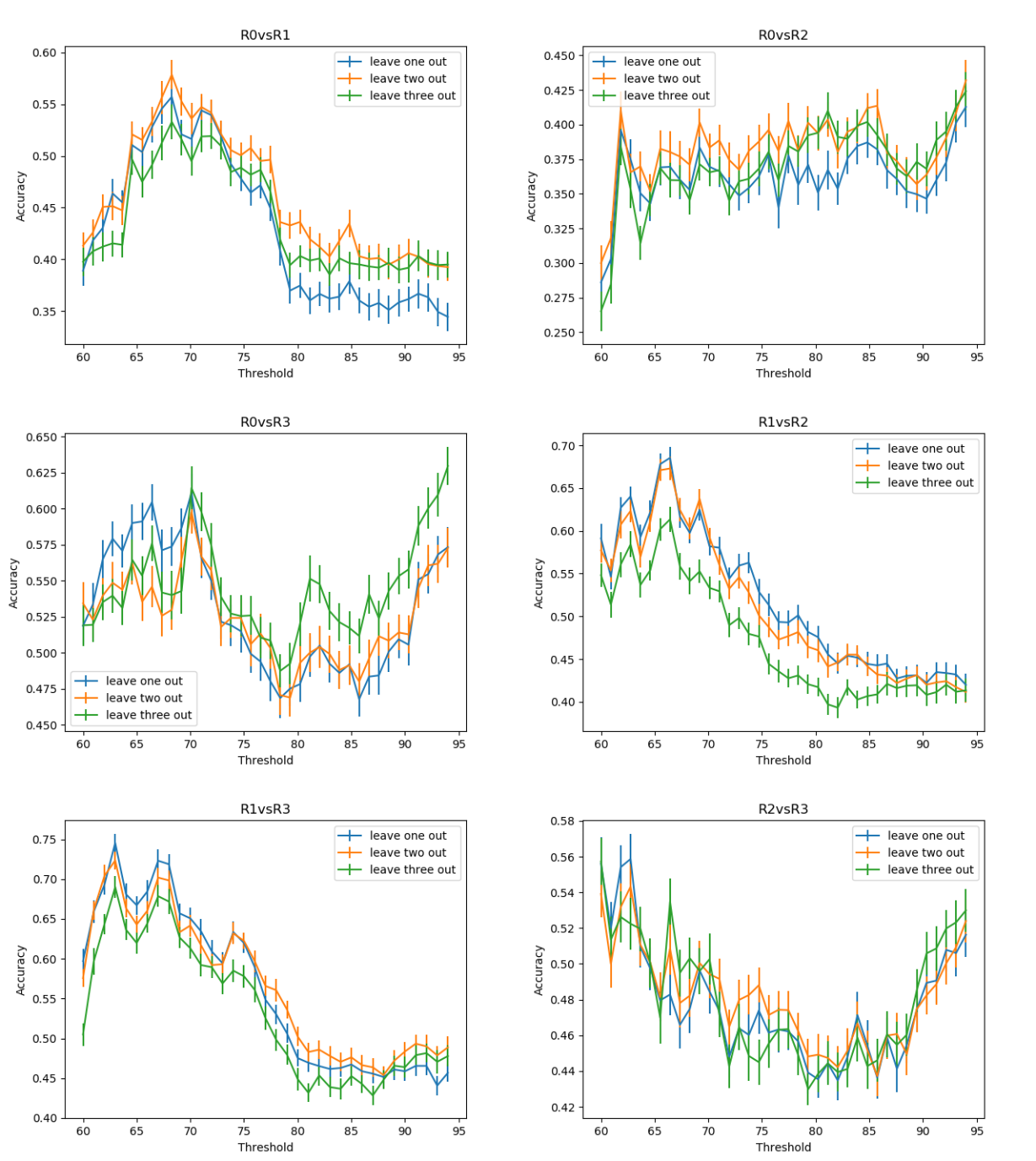
***

**Supplementary Figure 4:** Pairwise classification accuracies of boundary maps using three different cross-validation (CV) strategies


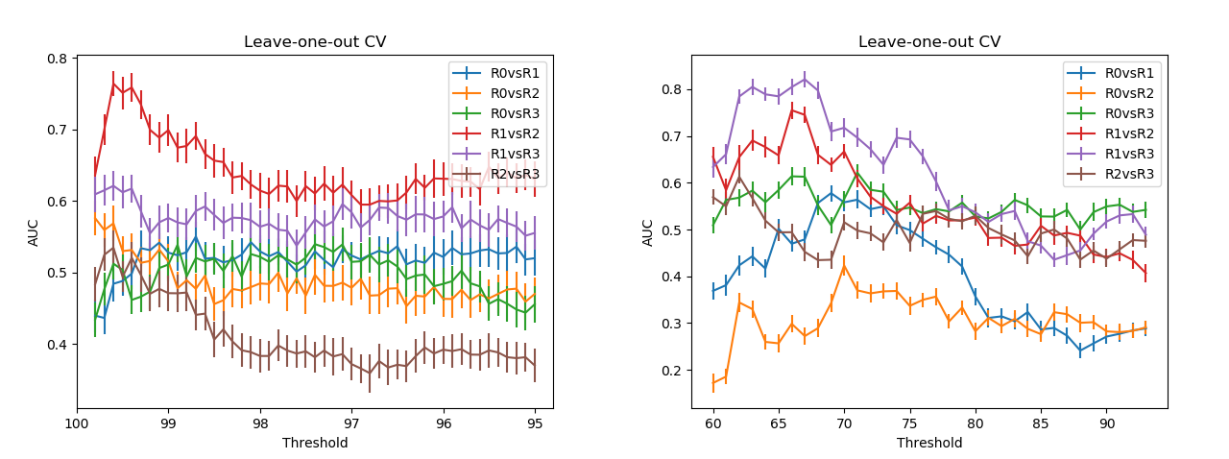


**Supplementary Figure 5:** Pairwise classification displayed as area under the curve (AUC) for node (left) and boundary mapping (right)

|  | Top ranking | Number of voxels | Accuracy [%] (95% CI) | AUC [%] (95% CI) |
| --- | --- | --- | --- | --- |
| Snowballing peak density | R1 vs R2 | 764 | 74.2 (73.0 – 75.4) | 76.4 (74.6 – 78.1) |
| Boundary mapping | R1 vs R2 | 346 | 68.5 (67.2 – 69.8) | 74.5 (72.7 – 86.3) |
|  | R1 vs R3 | 174 | 74.5 (73.4 – 75.6) | 80.5 (78.7 – 82.2) |

**Supplementary Table 1:**  Highest classification accuracies and area under the curve (AUC) for leave-one-out cross-validation (CV) of nodes and boundaries

**Supplementary Table 2:** Classification accuracies (%) on head mean frame-to-frame d
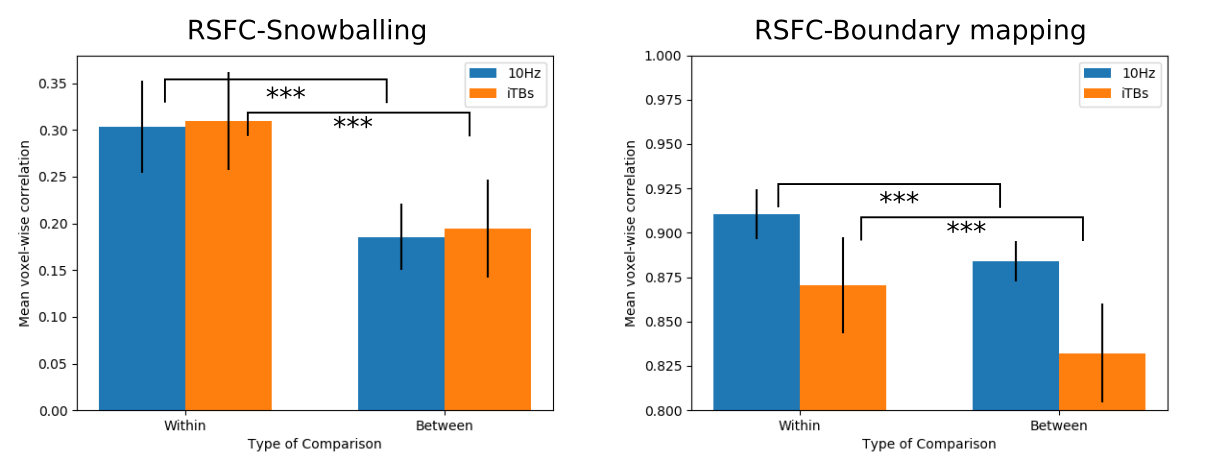
isplacement of real and sham rTMS

| Condition | Real  Mean (SD) | Sham  Mean (SD) | Real – Sham  Mean (SD) |
| --- | --- | --- | --- |
| R0 vs R1 | 0.50 (0.15) | 0.50 (0.29) | 0.57 (0.27) |
| R0 vs R2 | 0.52 (0.10) | 0.54 (0.20) | 0.52 (0.31) |
| R0 vs R3 | 0.52 (0.10) | 0.50 (0.00) | 0.35 (0.23) |
| R1 vs R2 | 0.54 (0.14) | 0.54 (0.20) | 0.46 (0.20) |
| R1 vs R3 | 0.57 (0.17) | 0.54 (0.14) | 0.52 (0.23) |
| R2 vs R3 | 0.50 (0.00) | 0.50 (0.00) | 0.50 (0.00) |

**Supplementary Table 3:** Significantly modulated regions from nodal and boundary maps for every comparison.

|  | Snowballing Peak Density | | Boundary Mapping | |
| --- | --- | --- | --- | --- |
| Comparison | **Brain region** | **Number of voxels** | **Brain region** | **Number of voxels** |
| R0 vs R1 vs R2 vs R3 | Angular_R | 104 | Occipital_Inf_L | 10 |
|  | Frontal_Mid_L | 65 | Precuneus_L | 28 |
|  | Insula_L | 107 | Precuneus_R | 10 |
|  | Insula_R | 83 | Temporal_Inf_R | 11 |
|  | Lingual_L | 55 |  | |
|  | Lingual_R | 78 |  |  |
|  | Occipital_Inf_R | 145 |  |  |
|  | Precuneus_L | 103 |  |  |
|  | Precuneus_R | 64 |  |  |
|  | Temporal_Sup_L | 70 |  |  |
|  | Temporal_Sup_R | 51 |  |  |
| R1 vs R2 | Angular_R | 21 | Calcarine_L | 11 |
|  | Insula_L | 21 | Precuneus_L | 17 |
|  | Insula_R | 18 | Temporal_Inf_R | 12 |
|  | Lingual_L | 20 |  | |
|  | Lingual_R | 17 |  |  |
|  | Occipital_Inf_R | 38 |  |  |
|  | Precuneus_L | 14 |  |  |
|  | Precuneus_R | 24 |  |  |
|  | Temporal_Sup_L | 13 |  |  |
| R1 vs R3 |  | | Calcarine_L | 13 |
|  |  |  | Occipital_Inf_L | 12 |
|  |  |  | Occipital_Mid_R | 17 |
|  |  |  | Precuneus_L | 24 |
|  |  |  | Temporal_Inf_R | 19 |
